# Electrospun nanofibers: building blocks for the repair of bone tissue

**DOI:** 10.3762/bjnano.15.77

**Published:** 2024-07-25

**Authors:** Tuğrul Mert Serim, Gülin Amasya, Tuğba Eren-Böncü, Ceyda Tuba Şengel-Türk, Ayşe Nurten Özdemir

**Affiliations:** 1 Ankara University, Faculty of Pharmacy, Department of Pharmaceutical Technology, 06560 Ankara, Turkey; 2 Erciyes University, Faculty of Pharmacy, Department of Pharmaceutical Technology, 38280 Kayseri, Turkey; 3 Istanbul Aydın University, Faculty of Pharmacy, Department of Pharmaceutical Technology, Istanbul, Turkey

**Keywords:** bone regeneration, controlled release, drug delivery, electrospinning, nanofibers

## Abstract

Bone, one of the hardest structures of the body, is the basic constituent of the skeletal system, which gives the shape to the body, provides mechanical support for muscles and soft tissues, and provides movement. Even if there is no damage, bone remodeling is a permanent process to preserve and renew the structural, biochemical, and biomechanical integrity of bone tissue. Apart from the remodeling, bone healing is the highly complicated process of repairing deficiencies of bone tissue by the harmonious operation of osteoblasts, osteocytes, osteoclasts, and bone lining cells. Various materials can be used to both trigger the bone healing process and to provide mechanical support to damaged bone. Nanofiber scaffolds are at the forefront of these types of systems because of their extremely large surface area-to-volume ratio, small pore size, and high porosity. Nanofibers are known to be highly functional systems with the ability to mimic the structure and function of the natural bone matrix, facilitating osteogenesis for cell proliferation and bone regeneration. Electrospinning is an easy and fast method to produce non-woven structures consisting of continuous ultrafine fibers with diameters ranging from micrometers down to nanometers. The simplicity and cost-effectiveness of the electrospinning technique, its ability to use a wide variety of synthetic, natural, and mixed polymers, and the formation of highly porous and continuous fibers are the remarkable features of this method. The importance of nanofiber-based scaffolds in bone tissue regeneration is increasing because of suitable pore size, high porosity, osteoinduction, induction of bone growth with osteoconduction, adaptability to the target area, biodegradation, and appropriate mechanical properties, which are among the main parameters that are important in the design of polymeric bone grafts. The aim of this review is to cast light on the increasing use of nanofiber-based scaffolds in bone tissue regeneration and give an insight about bone regeneration, production techniques of the electrospun nanofibers, and varying formulation parameters in order to reach different drug delivery goals. This review also provides an extensive market research of electrospun nanofibers and an overview on scientific research and patents in the field.

## Introduction

The nanofiber technology is a recent technology developed for producing implantable systems that can be used for structural support to the bones as well as drug delivery systems [1–5]. Because of the structural properties of nanofibers, which enable cell growth and proliferation, their use in tissue engineering, especially regarding bone tissue, is quite common [2]. Nanofiber scaffolds may carry active substances such as cells for tissue repair, antibiotics, anticancer agents, proteins, DNA, RNA, and growth factors for tissue regeneration [6–8]. In addition, nanofibers as drug delivery systems provide rapid or delayed and controlled release of pharmaceuticals.

Apart from being implantable drug delivery systems, nanofiber scaffolds can contribute to the healing process through their porous and flexible three-dimensional structure. They can increase the permeability of gases and liquids and reduce infection by bacteria because of their high filtration efficiency. Also, there is the great possibility of adding other functional moieties into the nanofibers.

## Review

### The architecture of the bone tissue

Bone, one of the hardest structures of the body, is the basic constituent of the skeletal system, which gives the shape to the body, provides mechanical support for muscles and soft tissues, and provides movement. Apart from these functions, the storage of calcium and phosphate and the protection of bone marrow are among the characteristics of bone tissue [9–10].

Bone tissue, which is a special form of connective tissue, consists of cells, fibers, and extracellular matrix; histologically, it can be divided into two bone types, namely, woven bone and lamellar bone. Woven bone is a transitional bone type observed in embryonic skeletons or growing bones, as well as during pathological conditions such as fracture repair or tumor. It gradually transforms into lamellar bone during the remodeling process in normal development or healing. In this sense, lamellar bone is secondary bone created by remodeling woven bone, and it is comprised of a series of lamellae. Nevertheless, it is the main type of bone in the healthy mature skeleton [11–12].

Lamellar bone consists of two different tissues differing in their structure, that is, dense tissue called compact or cortical bone and spongy tissue in a network structure consisting of thin rods and plates. This network structure allows for the formation of holes called trabeculae, which are interconnected, and this type of porous bone is known as cancellous bone or trabecular (spongy) bone. Compact bone forms the outer surface of all bones; it gives strength, provides great resistance to external forces, and determines the shape of the bone. In contrast, cancellous bone is mostly found at the ends of long bones and forms the inner part of the compact bone. Cancellous bone is quite strong and supports compact tissue despite its porous structure. One of the most important functions of cancellous bone is to allow for deformation and to absorb loads [9,13]. Also, the spaces between the trabeculae are the regions of the bone marrow where blood cells are formed; thus, this structure is responsible for supporting and protecting the red bone marrow.

In order to understand the regeneration process of bone, it is important to understand the structural arrangement of it. Both compact and cancellous bone tissue consist of hierarchical structural units at different levels [14]. Compact bone is organized through osteons (i.e., Haversian system), which are its most basic structural units. Osteons are cylindrical tunnels that run parallel to the long axis of the bone and consist of a calcified column filled with intracellular fluid. The Haversian canals are located in the middle of each osteon; they house nerves, lymphatics, as well as the blood vessels that are responsible for the transport of nutrients necessary for the maintenance of bone cells and tissues. The hierarchical organization and the multichannel structure of bone tissue support both nutrition and metabolism, increase bone strength, and help bone resistance [13–15]. It is also assumed that an increase in the porosity of compact bone due to enlarged canals or an increased number of canals may be associated with increased bone fragility [16].

Unlike compact bone, cancellous bone does not have a Haversian system. Instead, an irregular porous network structure is observed [17]. The porous structure of cancellous bone offers a high surface area and, therefore, provides ease of movement due to its low density. It is also more elastic than compact bone. Besides, with the help of the trabecular architecture of thin rods and plates, cancellous bone may support greater mass. Moreover, the metabolic turnover rate of cancellous bone is higher than that of compact bone; therefore, it is more active and has the potential to be rebuilt faster. Also, it acts as a reservoir in regulating the concentration of calcium and other mineral ions in the body [11–13].

In addition to all these structural units of bone, the outer surface of bones is covered with a thin fibrous membrane called the periosteum. The periosteum is a well-vascularized tissue, containing many blood vessels that penetrate the bone to nourish the bone cells and surrounding muscle tissue as well. It plays an important role in bone growth and fracture healing [18–19].

### The composition of bone tissue

Any bone in the body has both an organic and inorganic composite structure. The composition of extracellular bone matrix can be estimated as approximately 60–65% organic matrix, 20–30% inorganic components, and 10–15% water [13,18]. The organic matrix is responsible for the elasticity of the bone, while the inorganic matrix provides hardness. This means that the organic matrix gives bone its tensile strength, and the inorganic matrix improves the compressive strength of bone [16]. The most dominant component of the organic matrix is collagen, which is synthesized by osteoblasts and performs many mechanical functions. Collagen is a protein found abundantly not only in bones but also in almost every tissue of mammals. One third of all body proteins are collagens [20]. Type-I collagen constitutes approximately 90–95% of the organic matrix in bone tissue, and smaller amounts of other collagen types (i.e., III, V, X, and XII) are included in the bone composition [12,21]. It has been emphasized that type-I collagen is the most important protein structure that carries stress in mammals [20]. Tropocollagen, the most basic unit of collagen, is formed by the combination of three polypeptide chains, and each chain contains about 1000 amino acids. Type-I collagen forms well-organized fibers and fibrils. Collagen molecules in a triple helix structure self-assemble to form collagen fibrils, which, in turn, self-assemble to form collagen fibers. When collagen fibers, which are structural elements in bone, combine with inorganic materials, hardness and strength of the tissue are increased. Apart from collagen, the organic matrix of bone tissue mainly consists of osteocalcin, which is closely related to mineralization, other non-collagenous organic proteins such as osteonectin and osteopontin, as well as proteoglycans [10,12,22].

### The cellular structure of bone tissue

**Figure 1:**
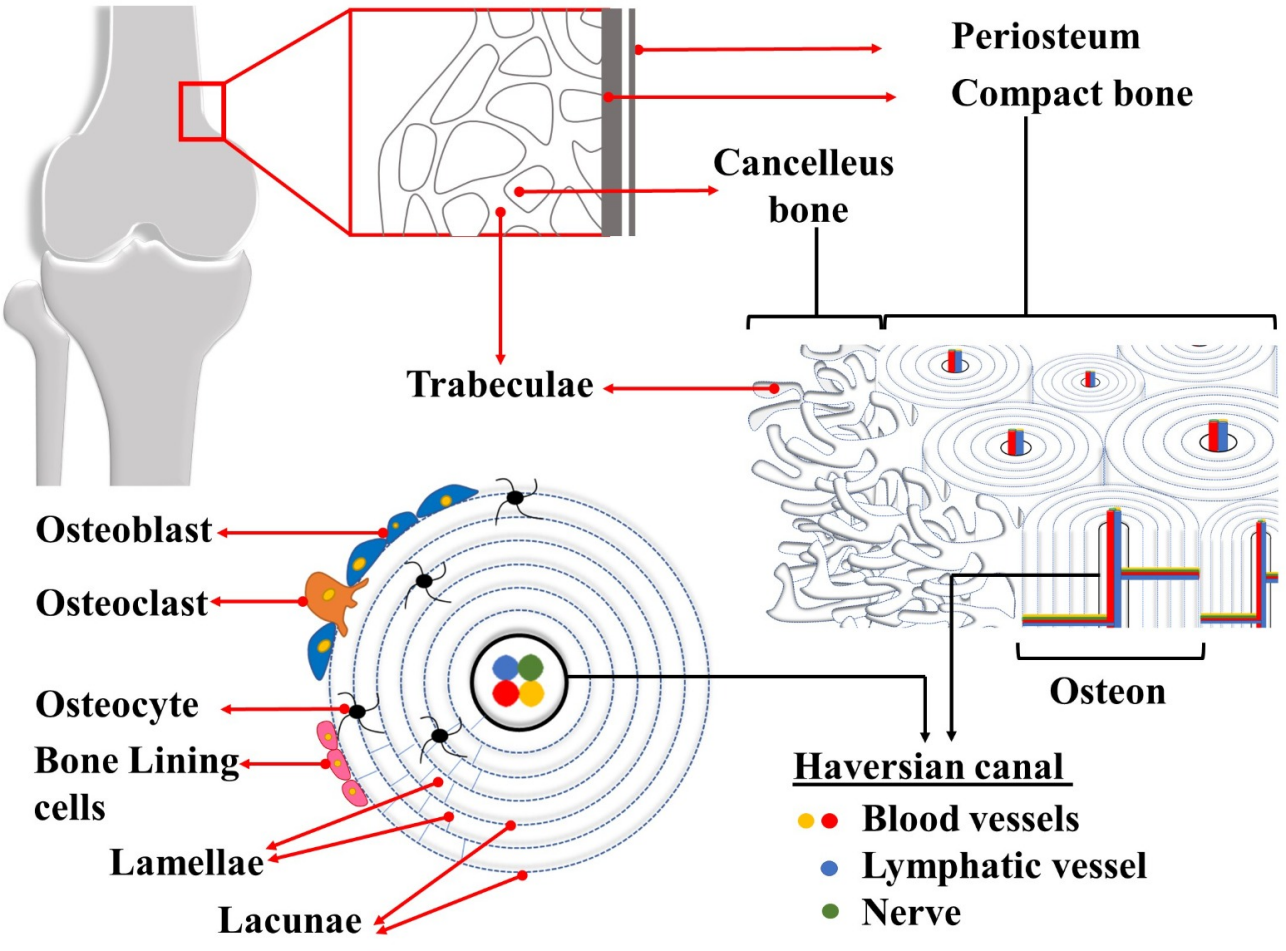
Illustration of bone tissue.

Bone, a living tissue, is constantly changing with the help of cells playing different roles. There are a number of specialized cell types that perform different functions for bone homeostasis, namely, osteocytes, osteoblasts, and osteoclasts. It has been stated that bone cells make up about 10% of the total bone volume [10,13]. The majority of bone cells are composed of osteocytes. Osteocytes derive from osteoblasts that have completed their task in bone formation. They are embedded in the organic matrix and they are designed to communicate directly with each other [23]. A mature osteocyte is surrounded by mineralized bone. The main function of an osteocyte is to sense mechanical stress and to convert it to a biochemical response. Osteocytes regulate bone remodeling by transmitting these signals to osteoblasts and osteoclasts. In addition, they play a role in the regulation of both local and systemic mineral metabolism [14,18,21]. In simplest terms, osteoblasts and osteoclasts are the two primary bone cell types responsible for bone remodeling. Osteoblasts are tightly bound to each other and they cover the bone surface. They are also known as protein-synthesizing cells and responsible for bone formation because they secrete organic bone matrix. Following the formation of this unmineralized cell matrix called osteoid, the mineralization phase begins, and new bone tissue is formed [9,14]. The main function of osteoclasts, which have properties similar to those of macrophages, is to resorb the mineralized bone matrix [13,18]. Another group of cells derived from osteoblasts are bone-lining cells, which cover the bone surface. Bone-lining cells remain inactive until a signal is received for bone growth or repair [14,21]. A schematic illustration of bone tissue is presented in Figure 1.

### Bone remodeling and bone healing

Even if there is no damage, bone remodeling is a permanent process to preserve and renew the structural, biochemical, and biomechanical integrity of bone tissue. To this end, osteoblasts and osteoclasts coordinate each other’s activities and keep bone resorption and bone formation in balance to maintain skeletal health. Depending on daily activities, microlevel damage may happen to bone. More serious bone damage may occur because of infectious diseases, congenital malformation, osteoporosis, or tumors; also, bone fractures may be the result of trauma. Bone healing is a highly complicated process of repairing the deficiencies of bone tissue by the harmonious operation of osteoblasts, osteocytes, osteoclasts, and bone lining cells [24]. Actually, there are two basic mechanisms, namely, direct and indirect bone healing. Direct healing without callus formation is rarely seen in the natural healing process. Therefore, indirect healing is to be detailed here [25]. In general, three successive stages, that is, inflammatory, repair, and bone remodeling stages, are observed in bone healing or repair [26]. Bone damage is often accompanied by damage of both periosteum and surrounding soft tissue. In the first stage of healing, a hematoma is immediately formed by the migration of blood cells to the damaged area, and an increase in local tissue volume is observed. The purpose of hematoma formation is to gain temporary mechanical stability and to protect the area during the following healing phases. At the same time, an immediate pro-inflammatory cytokine release occurs to create an inflammatory reaction. In the repair phase, where the hematoma is replaced by fibrin-rich granulation tissue over time, the granulation tissue first evolves into fibrous tissue consisting of fibroblasts and then into soft callus. At this stage, new vessels, fibroblasts, intracellular material, and supporting cells begin to be produced, and nutrition, oxygen, growth factors, and other components for fracture healing are provided [27]. With the calcification of the soft callus, the hard callus is shaped, which is strong enough to hold damaged bone tissue together. Finally, compact bone tissue is formed with the help of increased osteoblast/osteoclast activity, and remodeling is observed by resorption and formation of new bone tissue. Unlike other tissues, scar tissue is not observed in most bone injuries, and healing occurs through the formation of new bone tissue [27–28].

Various materials can be utilized in order to both trigger the bone healing process and provide mechanical support to damaged bone. Currently, research on bone healing has focused on the development of biomaterials that can be used as economic, biocompatible, and controllable bone substitutes. Studies have centered on biomaterials that can imitate natural bone structure with appropriate porosity and fulfill the functions of transport as well as the exchange of substances. Biomaterials should also help cells to adhere and maintain their normal proliferative and differentiation capacity. Nanofiber scaffolds are at the forefront of these types of systems because of their extremely large surface area-to-volume ratio, small pore size, and high porosity. Nanofibers are known to be highly functional systems with the ability to mimic the structure and function of the natural bone matrix and to facilitate osteogenesis for cell proliferation and bone regeneration [25,29].

### Polymeric nanofibrous scaffolds

Bone has the ability to heal after fractures and to regenerate continuously. Nevertheless, tumor resections, traumatic bone loss, or large defects caused by infections cannot heal without surgical interventions, and treatments targeting skeletal complications are a major challenge in the field of orthopedics. In order to both trigger the bone healing process and provide mechanical support to the damaged bone, synthetic scaffolds with nanofibrous structure have been investigated recently.

Polymeric nanofibers are nanostructured systems that can also be used as drug delivery systems, which are composed of fibers from a few micrometers down to tens of nanometers in diameter [30]. Among the various scaffolds used for bone regeneration, polymeric nanofibrous scaffolds have attracted attention in recent years, especially regarding their structural superiorities. Their unique characteristics that distinguish them from other scaffolds and pharmaceutical forms also constitute their main advantages: (i) Their network nature mimics the natural extracellular matrix. In this way, nanofibrous scaffolds provide a higher level of cell adhesion, differentiation, migration, and proliferation than other scaffolds, especially particulate forms [31–32]. (ii) Their large surface area per unit volume, adjustable high porosity, and superior flexibility and mechanical properties enable them to adhere to bone tissue more easily, to carry biofactors such as growth factors, and to release these factors in a controlled manner [33]. (iii) Their non-toxic and biodegradable structures ensure that they are compatible with the body. These properties also allow for the delivery of a wide range of active molecules such as antibiotics [34]. (iv) The possibility to functionalize the surface of the nanofibrous scaffolds increases the therapeutic response to the drugs by a controlled and sustained release in the targeted tissue [35]. (v) Their ability to carry different drugs in their structure reduces the risk of multidrug resistance in cancer treatment with dose-specific or site-specific release of various active molecules [35]. (vi) Their nanometer dimensions yield superiority over traditional solid membranes, such as ease of use, packaging, and transportation [8].

### Nanofiber production methods

There are several production methods for fiber-based scaffolds mentioned in the literature including inter-surface polymerization, phase separation, drawing, self-assembly, melt blowing, template melt extrusion, forcespinning, and electrospinning [36–40]. Only electrospinning will be further explained in detail in order to remain within the scope.

#### Electrospinning method

Electrospinning is an easy and fast method used to produce non-woven structures consisting of continuous ultrafine fibers with diameters ranging from micrometers down to nanometers. In this method, nanofibers are produced from a polymer solution or a melt with the help of electrostatic forces [41]. The simplicity and cost-effectiveness of the electrospinning technique, its ability to use a wide variety of synthetic, natural, and mixed polymers, and the formation of highly porous and continuous fibers are the remarkable features of this method [42].

Although the use of electrospinning has become widespread with the developments in nanotechnology after the 1980s, the foundations of this method are much older. Electrospinning was first tried by Rayleigh in 1897, upon William Gilbert's discovery that fluid dynamics was influenced by electrical fields in the 16th century. In the early 1900s, Zeleny examined this method in detail. The first patent for turning a polymer solution into nanofibers by electrostatic forces was issued to J. F. Cooley in 1902. Between 1934 and 1944, Anton Formhals further developed the electrospinning method and equipment and obtained new patents [43]. In the 1990s, especially the studies of Reneker and his working group on the production of fine fibers from various organic polymers contributed significantly to the widespread use of electrospinning for the preparation of nanofibers today [44].

**Figure 2:**
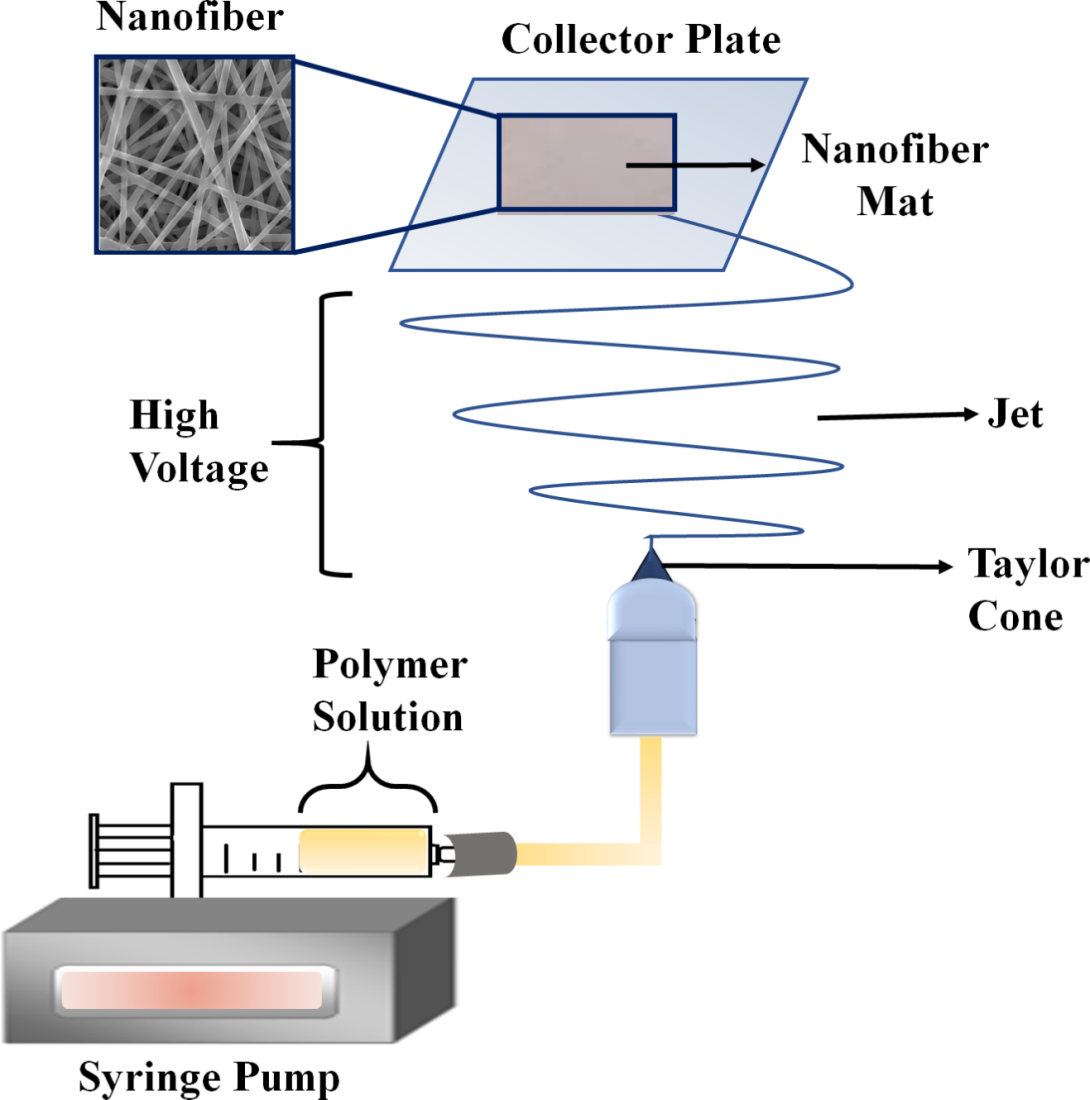
Schematic representation of the electrospinning process.

A schematic representation of the electrospinning process is given in Figure 2. There are basically four main elements in an electrospinning assembly. These are (i) a high-voltage power source, (ii) a feeding unit (i.e., nozzle, syringe, or metal needle), (iii) a collector (i.e., plate, cylinder, disc, or rotating drum), and (iv) a viscous polymer (melt or solution) in liquid form [44]. The polymer solution in an appropriate solvent is placed into a syringe for the electrospinning procedure. The plate positioned at a specific distance is connected to the high-voltage source. The polymer solution forms a Taylor cone, which is a conical structure at the needle tip, when an increasing electric field (1–30 kV) is supplied to the syringe tip [45]. A polymer jet exits the Taylor cone structure when the electric field applied to the polymer solution rises above a predetermined threshold. High-voltage-polarized polymer molecules travel in an erratic manner in the direction of the motion axis, resulting in a disorganized network structure on the collector plate. As the jet moves towards the collector, stretching and bending occur depending on the propellant loads along the jet [46].

“Single needle” is the simplest method used at the laboratory level for nanofiber production. With a coaxial needle design, two different solutions can be electrospun simultaneously, and core–shell or hollow fibers can be formed [47]. The main parameters affecting fiber morphology are described in the following.

#### Process parameters

Flow rate, voltage, distance, collector type, concentration and molecular weight of the polymer, solvent or solvent mixture, surface tension, conductivity, dielectric constant, and environmental parameters are parameters that must be optimized in the electrospinning process.

#### Voltage

Voltage is the parameter that most affects the shape of the droplet from which the jet at the needle tip is formed [48]. When the voltage reaches a critical value, the polymeric fluid forms a Taylor cone. The Taylor cone is not formed, and nanofibers cannot be produced by electrospinning, below the critical voltage value. Overvoltage causes the rate of removal of the solution from the needle tip to be higher than the rate required for Taylor cone formation [49–50]. Thus, it may cause multiple jets or destabilization of the Taylor cone, resulting in an increase in bead defect density [49,51–52]. The fiber diameter decreases with increasing voltage [53]. The bead defect density increases above a critical voltage [49]. The aspect ratio of the obtained beads increases up to a certain voltage value and then decreases [50,54].

#### Flow rate

Insufficient flow rate does not allow for jet formation. In contrast, excessive flow rates will cause an increase in the polymer liquid accumulating at the needle tip above the stretching ability of the electric field strength; hence, polymer liquid may flow from the needle tip [49,52,55]. Generally, as the flow rate increases, thicker and more porous fibers with a wide distribution are formed [56–57]. When the flow rate is too high, beaded nanofibers and many other defects are formed mainly because of more unspun droplets as a result of larger droplet volume and increased bending instabilities [56,58].

#### Needle tip-to-collector distance

Since the distance between needle tip and collector influences the amount of evaporated solvent, it affects the fiber formation and the diameter/morphology of the obtained fibers. Therefore, the distance for electrospinning needs to be optimized [59]. Electrospraying can be observed when the distance is too short or too long. Also, if the distance is too short, the solvent of the polymer solution cannot evaporate before it reaches the collector, which causes the fibers to adhere to the collector and to coalesce [60–61].

Also, when the distance is too short or too long, beaded structures form [59]. The aspect ratio of the obtained beads decreases with increasing needle tip-to-collector distance [50]. A decrease in the distance causes an increase in the electric field strength and causes the fibers to gather on the collector plate without thinning and elongation; thus, larger nanofiber diameters are obtained [44,52,62].

#### Collector type (rotating/fixed)

A collector rotating at a certain speed allows for the production of aligned nanofibers, while a fixed collector generally produces non-aligned nanofibers. Also, a rotating collector leads to thinner fibers [63–64]. By changing the rotation speed, the alignment and the diameter of the fibers can be changed [64].

#### Polymer concentration

The polymer concentration is one of the most important parameters for electrospinning as it affects solution viscosity. When the concentration/viscosity is low enough, electrospraying is observed instead of electrospinning. If the concentration/viscosity is excessive, the polymer fluid freezes at the needle tip and there is neither electrospraying nor electrospinning. So, optimizing the polymer concentration is essential for stable Taylor cone formation [4,55].

As the concentration/viscosity increases, smoother nanofibers with larger diameter are generally formed, while number and size of the beads generally decrease. By increasing the polymer concentration to an optimum level, beads can be completely eliminated [4,55,65]. The aspect ratio of the beads increases with increasing polymer concentration [50,54]. At higher concentrations, as the number of polymer chains per unit volume of solvent increases, entanglement and interaction of polymer chains will occur to a greater extent, resulting in an increase in elastic properties of the fibers [54].

#### Polymer molecular weight

The molecular weight of the polymer is another important parameter regarding electrospinning as it affects solution viscosity by causing a change in the density of polymer chains in the solution. It is essential to determine the ideal polymer concentration for homogeneous fiber formation [62]. With an increase in molecular weight and viscosity of the polymer, larger fiber diameters, smoother nanofibers, and fewer beads are observed. Pore size and porosity percentage are also related to the molecular weight of the polymer [57,66].

#### Solvent or solvent mixture

The solvent, or the solvent mixture, used is critical for the stable formation of a Taylor cone. It affects the intermolecular interaction in the polymer–solvent system and causes a change in viscosity, surface tension, and electrical conductivity of the polymer liquid [54,65]. The evaporation rate of the solvent is an extremely important parameter. In an ideal production process, the solvent must be completely evaporated before the jet reaches the collector of the device. If the solvent does not evaporate completely, the formed nanofibers will remain wet and tend to coalesce. In contrast, if the solvent evaporates too quickly, proper jet elongation may not occur since a flow at the appropriate speed may not be achieved. This will cause an increase in the diameter of the nanofiber. Therefore, shorter spinning distances should be used when working with highly volatile solvents [35]. The most commonly used solvents in electrospinning polymeric nanofibers are methanol, acetone, acetic acid, dichloromethane, dimethylformamide, ethyl acetate, trifluoroethanol, ethanol, formic acid, and tetrahydrofuran. Depending on the polymer type or the hybrid polymer composition, these organic solvents can be used alone or applied as mixtures in order to obtain suitable solution properties [67].

#### Surface tension

The surface tension of the polymer liquid is important for the electrospinning process because a stable Taylor cone can only form when the electric potential is high enough to overcome the surface tension of the solution [51,68–70]. The surface tension of the polymer liquid directly affects the critical stress; with increasing surface tension, the critical voltage required for Taylor cone formation increases [50]. With the decrease in surface tension, fewer beads are formed [55,71], and ionic surfactants can be used to reduce the number of beads by the lowering surface tension [72–73].

#### Conductivity

The conductivity of the solution is of great importance to improve the spinnability. It should be high enough for electrospinning. The polymer liquid is stretched by the charges collected on the drop at the needle tip for electrospinning process. If the conductivity of the solution is not sufficient, a jet cannot form, or a homogeneous fiber cannot be produced as the polymer liquid will not be fully stretched [74–75]. Salts or surfactants can be added to increase the conductivity [76–78]. Optimizing the conductivity is essential to prevent bead formation and to improve fiber structures [76–78]. With the increase of conductivity smaller fiber diameters and fewer beads are formed [79].

#### Dielectric constant

The dielectric constant refers to the ability of a substance to hold an electric charge in the region where an electric field is applied. The dielectric constant of a polymer liquid mainly depends on the solvent used. For the electrospinning process, the dielectric constant must be high enough [74–75]. With an increase in dielectric constant fewer beads and smaller fiber diameters are formed [80].

### Environmental parameters

#### Temperature

Temperature affects the viscosity and spinnability of polymer liquids and the thickness of the collected fiber mats. Controlling the temperature influences the electrospinning process and is important for reproducibility [81–82]. The morphology of the fibers is affected by temperature. With the increase in temperature, smaller fiber diameters and smoother fibers are observed [81,83]; also, viscosity decreases with increasing temperature [84].

#### Humidity

Stable humidity during electrospinning is crucial for a high reproducibility of the fibers [85]. With increasing humidity, the concentration of dissociated ions in the fibers and charge repulsions among the fibers increase during electrospinning [86]. If the humidity is high, water may accumulate on the fiber surfaces, and if the humidity is too low, the solvent may evaporate too quickly [73,82]. Also, relative humidity makes nanofibers thicker or thinner depending on the chemical structure of the polymer [82].

#### Drug release from electrospun nanofibers

The rate and the mechanism of release of active material from the nanofibers can be adjusted by changing the type and composition of polymer or polymer blends used as matrix and the process parameters. Some of the parameters affecting the release are (i) degradation of the polymer matrix, (ii) molecular weight of polymer and drug, (iii) hydrophilicity/hydrophobicity of polymer and drug, (iv) properties of additives, (v) morphology of the system (e.g., porosity), and (vi) drug loading [4]. The aim of nanofiber production may be to provide the release of active material with zeroth-order kinetics after the burst effect at the initial stage through the release of active material directly on the surface of the nanofibers. Extended release is also possible, which is provided by diffusion through the polymeric nanofiber [35]. The burst effect creates the first effective concentration in the targeted area [87–90]. In cases where the burst effect is not desired, hydrophobic polymer blends need to be used. Furthermore, either core–shell or laminated nanofibers can be produced [32,91]. The degradation of polymers, the diffusion of the active material, or both of them may affect the extended release phase. The polymer may degrade during or after the release of active material by diffusion. The in vivo degradation times for commonly used polymers change from days to months [52,61,92]. Different properties of the polymers lead to a wide range of degradation and drug release rates [33,93]. Since the regeneration of damaged bone tissue can take several weeks or even months, it is important to select a polymeric matrix capable of releasing drugs over an extended period of time to achieve optimum therapeutic efficiency. The polymer must also be chosen in accordance with the targeted release mechanism. It is difficult to achieve the desired release rate in systems in which both diffusion and erosion occur. Preferably, the polymer should degrade after the release of the active material. A high solubility of the active material in the polymer increases the rates of diffusion and release. If the active ingredient is dissolved in the polymer, Higuchi homogeneous matrix kinetics is observed, and the active substance passes through the matrix by diffusion. Higuchi heterogeneous matrix kinetics also plays an active role in the release when an excess of active substance is present in the polymer. Water-soluble additives in the nanofiber matrix cause an increase in the release of active material through the water-filled pores in the system [4,94–95]. Pores are created in the eroded polymeric structure and accelerate the release rates [32].

#### Formulation components of polymeric nanofibers

The basic components of bone tissue are 60% organic matrix, 30% mineralized inorganic matrix, and 10% water. About 90–95% of the organic matrix is type-I collagen, which is the main component of connective tissue. About 70% of the mineralized part of the bone that forms the inorganic matrix component is hydroxyapatite. Hydroxyapatite affects hardness and resistance of bone tissue. Mechanically, the organic part provides flexibility of the bone tissue, while the inorganic part ensures the hardness and a strong and solid structure. Therefore, calcification of artificial bone grafts is essential for the supply of hard bone tissue. Hence, polymers are the most important formulation components in the preparation of nanofibers with a structure similar to natural bone tissue for bone regeneration [96]. The content and alignment of the polymeric structure of the nanofibers is important for the alignment of the collagenous fibers in hard bone-like tissues. Calcified bone tissue shows different mechanical properties depending on the collagen sequence in the natural structure. The polymers used in the development of polymeric nanofibers should have mechanical and biological properties suitable for calcified hard tissue structures [33]. In addition, given their compatibility with various active molecules, the polymers are the most essential formulation components in providing long-term controlled release of drugs. Biodegradability, biocompatibility, hydrophilicity, and mechanical properties of polymers are important criteria when preparing polymeric nanofiber formulations to obtain controlled and sustained drug release profiles. The polymers used in the preparation of polymeric nanofibers are generally categorized into four classes, namely, natural polymers, synthetic polymers, blends of these two classes (hybrid), and inorganic materials [32]. Types, advantages, and limitations of polymers used for the production of polymeric nanofibers are given in Table 1.

**Table 1:** Summary of polymer types, advantages, and limitations [32,97–98].

Polymer class	Polymer type	Advantages	Limitations

natural	chitosan	- biocompatibility - non-toxic - similarity to the macromolecules in the human body - similarity to the extracellular matrix - commercial availability	- difference between batches - containing immunogenic/pathogenic parts - sensitive to cross contamination - difficulty being electrospun
synthetic	poly(lactic acid)	- safe usage - structures are well defined - easy to electrospun - physicochemical properties can be easily controlled - degredation and mechanical properties can be adjusted	- difficulty being recognized and attached by the cell
hybrid	collagen–hydroxyapatite	- high similarity to the extracellular matrix - degredation and mechanical properties can be adjusted - biocompatibility - biodegredability	- difficulty being electrospun - difference between batches
inorganic	hydroxyapatite	- high similarity to the extracellular matrix - biocompatibility - biodegredability	- difficulty being electrospun - difficulty in maintaining mechanical strength

Solvents are the most important component after polymers in polymeric nanofiber formulations. Solvents must not interact chemically with the polymer and must dissolve the polymer at the appropriate concentration for nanofiber formation [60].

#### Commercial products of electrospun polymeric nanofibrous scaffolds

The industrial production of polymeric nanofibers by electrospinning has paved the way for commercial products in many fields including tissue engineering, bone regeneration, dermal patches, antimicrobial wound care, and cardiovascular grafts. Many industrial-scale electrospinning apparatuses have been developed by different companies, such as NanospiderTM (Elmarco s.r.o.) [99], Nanospinner 416 (Inovenso) [100], and INFL8100 (Fanavaran Nano-Meghyas (FNM Co. Ltd.)) [101].

The main polymeric nanofiber products on the market for bone regeneration are ReBOSSIS (Ortho Rebirth, Japan), a PLGA-based biomedical product for filling a synthetic bone cavity, poly-ʟ-lactic acid (PLLA) and collagen scaffolds (The Electrospinning Company, United Kingdom), and PCL-based nanofiber scaffolds for tissue regeneration (Stellenbosch Nanofiber Company, South Africa) [45].

ReBOSSIS consists of β-tricalcium phosphate, PLGA, and silicon-doped calcium carbonate to support bone formation. ReBOSSIS electrospun fibers are distinguished from other market products by a cotton-like nanofiber structure and the formulation components. Thus, ReBOSSIS provides ease of use in filling different shapes and sizes of bone voids during operations. In addition, unlike granular artificial bone material, it does not fall out of the bone cavities after the operation. During the patients’ recovery, ReBOSSIS is gradually replaced with bone. ReBOSSIS was approved by the US Food and Drug Administration in April 2015, and it us available in the USA [102]. PLLA and collagen-containing scaffolds developed by The Electrospinning Company are used for biomedical applications in various bone defects. The nanofiber scaffolds developed for this purpose are in the form of random non-woven scaffolds and consist of highly aligned fiber membranes and combination scaffolds. The approximate porosity of such nanofibers is 80–95%, and the fibers are produced with a thickness of about 4 mm [103]. Nanofiber scaffolds containing poly(ε-caprolactone) developed by Stellenbosch Nanofiber Company are used in tissue regeneration [104].

#### Patents regarding polymeric nanofibers used in bone regeneration

The patent search database https://worldwide.espacenet.com/ of the European Patent Office lists 700 results for the search term “polymeric nanofiber bone regeneration”. Examples of patents for polymeric nanofiber structures patented for use in bone regeneration are tabulated in Table 2.

**Table 2:** Examples of patents for polymeric nanofiber structures patented for the use in bone regeneration [105].

Patent name	Publication number	Publication date

Biomedical patches with aligned fibers	US10617512B2 US2019365520A1	2019-12-05 2020-04-14
Methods of fabricating 3d hierarchical nanofiber scaffolds with structural and/or compositional gradients	WO2022020310A1	2022-01-27
Fiber-based scaffolds for tendon cell migration and regeneration	WO2021077042A1	2021-04-22
Composition and method for producing controlled release systems containing an agent for guided bone regeneration	WO2020255107A1	2020-12-24
Bone regeneration using biodegradable polymeric nanocomposite materials and applications of the same	US2017281829A1 CA2964107C	2019-03-26 2021-02-02
Method for preparing barrier membranes for tissue regeneration	WO2019157583A1	2019-08-22
Nanofiber paste for growth factor delivery and bone regeneration	WO2017066545A1	2017-04-20
Multi-component electrospun fiber scaffolds	AU2016254147A1 AU2016254147B2	2017-11-16 2020-09-03
Composite of nanofiber and hydrogel, and scaffold for tissue regeneration comprising same	WO2020204230A1	2020-10-08
Nanofiber microspheres and methods os use thereof	US2021212949A1	2021-07-15
Osteoinductive nanofiber scaffold for bone regeneration	WO2017201259A1	2017-11-23
Biomedical patches with spatially arranged fibers	US10682444B2 US2019365958A1	2019-12-05 2020-06-16
Systems and methods for repairing soft tissues	US2020022695A1	2020-01-23
Biomimetic nanofiber tissue scaffolds	US11147900B1	2021-10-19
Scaffold comprising carbon nanotube interfaced biopolymer nanofiber and preparation method thereof	KR20210069760A	2021-06-14
Chitosan nanofiber microsphere and preparation method thereof	CN113336977A	2021-09-03
Nanofiber structures and methods of manufacture and use thereof	CA3123236A1	2020-06-18
Fibrous polymeric scaffolds for soft tissue engineering	WO2021030229A1	2021-02-18
Biomimetic lamellar tissue scaffolds	US10876095B1	2020-12-29
Novel electrospun synthetic dental barrier membranes for guided tissue regeneration and guided bone regeneration applications	WO2019126819A1	2019-06-27
Guided bone regeneration barrier membrane	CN116392646A	2023-07-07
Device for the treatment of periodontal disease	ES1286510U	2022-02-11
Methods of fabricating 3D hierarchical nanofiber scaffolds with structural and/or compositional gradients	US2023302196A1	2023-09-28
In situ forming composite material for tissue restoration	US2023338612A1	2023-10-26
Hybrid, artificial bone tissue implant absorbing mechanical vibrations, whose architectural structure imitates trabecular bone, allowing the saturation of bone marrow, blood, and nutrients, supporting autological regeneration, which can be used with titanium structures	WO2022220766A1	2022-10-20
Composition and method for producing bioceramic, bioactive bone grafts and membranes made of synthetic hydroxyapatite	WO2023070178A1	2023-05-04

## Conclusion

Bone is a dynamic structure that has the ability to regenerate continuously and to heal after fractures. However, tumor resections, traumatic bone loss, or large defects caused by infections cannot heal without surgical interventions. Hence, treatments for skeletal complications cause difficulties in the field of orthopedics. In order to both trigger the bone healing process and to provide mechanical support to the damaged bone, research in recent years has focused on synthetic scaffolds to stimulate the natural healing process of bone.

Polymeric nanofiber scaffolds are of great interest in the fields of drug-release devices and tissue regeneration matrices. In bone tissue engineering, nanofiber scaffolds are widely used for bone regeneration. Small fiber diameter, high surface area-to-volume ratio, high porosity, and the nanoscale biological structure of the natural extracellular matrix give nanofibers superiority in the treatment of bone regeneration and increase the treatment efficiency. Polymeric nanofibers can be produced using various production techniques such as self-coupling and phase separation, and especially electrospinning. Electrospinning is a simple, scalable and versatile fabrication method for the production of various nanofiber formulations from a large number of different polymers. Polymer fiber diameter and morphology can be varied by varying the process and formulation parameters.

The importance of nanofiber-based scaffolds in bone tissue regeneration is increasing because of suitable pore size, high porosity, osteoinduction, induction of bone growth with osteoconduction, adaptability to the target area, biodegradation, and mechanical properties, which are among the main parameters important in the design of polymeric bone grafts.

## Data Availability

Additional research data is not shared.
